# Notice

**Published:** 1879-11-20

**Authors:** 


### EDITORIAL AND MISCELLANEOUS.

### *NOTICE.*

Parties desiring to subscribe for the RECORD for 1880 can commence with the November or December number without extra charge. Any one of our present subscribers on renewing their subscriptions for 1880 can secure club rates by sending up two new subscribers, and any one will be allowed a commission of 25 per cent. on all new subscriptions which may be sent us. Our Journal is constantly improving, and is now regarded as eminently practical, and as presenting more useful information in less space than perhaps any other Journal in the whole country. Such is the testimony that comes up to us from the profession in all sections.

